# ﻿Three novel species of *Alternaria* (Pleosporales, Pleosporaceae) from cereal crops (Poaceae) in China

**DOI:** 10.3897/mycokeys.116.145681

**Published:** 2025-04-16

**Authors:** Hai-Feng Liu, Feng-Yin Liu, Hai-Yan Ke, Qing-Xiao Shi, Jian-Xin Deng, Hyunkyu Sang

**Affiliations:** 1 Department of Plant Protection, College of Agriculture, Yangtze University, Jingzhou 434025, China Chonnam National University Gwangju Republic of Korea; 2 Department of Integrative Food, Bioscience and Biotechnology, Chonnam National University, Gwangju, 61186, Republic of Korea Yangtze University Jingzhou China

**Keywords:** Dematiaceous hyphomycetes, maize and rice diseases, morphology, new taxa, multigene phylogeny, taxonomy

## Abstract

The genus *Alternaria* (Pleosporales, Pleosporaceae) comprises saprophytes and pathogens that are widespread around the world. Currently, more than 400 species are recognized within this genus and are classified into 29 sections. In this study, *Alternaria* strains were isolated from diseased leaves of two cereal crops, rice (*Oryzasativa*) and maize (*Zeamays*) in China. These *Alternaria* spp. were characterized by morphological characterization and phylogenetic analysis using maximum likelihood and Bayesian inference with multiple loci (ITS, *GAPDH*, *RPB2*, *TEF1*, *Alt a 1*, *EndoPG*, and OPA10-2). Based on the above analyses, three novel species of AlternariasectionAlternaria were introduced, namely *A.oryzicola***sp. nov.**, *A.poae***sp. nov.**, and *A.zeae***sp. nov.** This study expands the species diversity of *Alternaria* associated with Poaceae plants in China.

## ﻿Introduction

The genus *Alternaria* consists of more than 400 species of dematiaceous hyphomycetes (Li et al. 2022, Gou et al. 2023, Liao et al. 2023, Hyde et al. 2024). Species in this genus have been mainly described as saprophytes, endophytes, or phytopathogens and currently accommodated in the family Pleosporaceae (Hyde et al. 2024). Historically, taxonomy of *Alternaria* has gone through different stages since it was first established by Nees in 1816 (Lawrence et al. 2016). In brief, *Alternaria* and related genera, especially *Macrosporum* and *Stemphylium*, were confused in earlier stages. Although attempts were made by researchers to determine their taxonomic status, issues in nomenclature and generic boundaries persisted for a long time. Afterwards, a complete revision of taxa related to *Alternaria* based on sporulation patterns and conidial morphology was undertaken by Simmons (Simmons 2007), which accelerated the establishment of order in the nomenclature of alternarioid hyphomycetes.

Nowadays, a DNA-based molecular approach has been used to better understand taxonomy of *Alternaria* (Lawrence et al. 2016). A variety of loci have been used in the classification of this genus, such as the internal transcribed spacer (ITS) of the rDNA region, small subunit ribosomal RNA gene (SSU), large subunit ribosomal RNA gene (LSU), glyceraldehyde-3-phosphate dehydrogenase (*GAPDH*), second largest subunit of the RNA polymerase (*RPB2*), translation elongation factor 1-α (*TEF1*), *Alternaria* major allergen (*Alt a 1*), endopolygalacturonase gene (*EndoPG*), an anonymous genomic region (OPA10-2), calmodulin (*CAM*), and the plasma membrane ATPase gene (*ATP*). Based on multi-locus phylogenetic analysis, this genus consists of 29 sections (Gannibal et al. 2022, Li et al. 2023). Currently, both morphology and multi-locus phylogeny are crucial for the taxonomy of *Alternaria* spp. and have been widely employed in charactering novel species (Aung et al. 2024; He et al. 2024; Nwe et al. 2024; Bessadat et al. 2025).

*Alternaria* spp. have been associated with more than 4,000 host plants, ranking the genus 10^th^ among the 100 most cited fungal genera (Lawrence et al. 2013; Woudenberg et al. 2013, 2015; Pinto and Patriarca 2017; Li et al. 2022; Bhunjun et al. 2024). Collectively, certain *Alternaria* spp. cause diseases that lead to economic losses in agricultural crops, including cereals, oil crops, fruits, and vegetables (Li et al. 2022; Haituk et al. 2023; Alasadi 2024). Cereal crops (Poaceae), such as wheat (*Triticumaestivum*), maize (*Zeamays*), and rice (*Oryzasativa*), have been widely cultivated and consumed since they are popular staple foods with most of the world’s population. Infections on cereal crops caused by *Alternaria* spp. occur constantly and have attracted increasing attention worldwide (Tralamazza et al. 2018; Orina et al. 2021; Zhong et al. 2022). For instance, wheat black point is an important disease mainly caused by different *Alternaria* species (*A.alternata*, *A.infectoria*, and *A.tenuissima*), with *A.alternata* being isolated more frequently (Pinto and Patriarca 2017; Tralamazza et al. 2018). In addition, *A.tricitina* has been considered as another important pathogen causing leaf blight on wheat (Mercado Vergnes et al. 2006; Amatulli et al. 2013). Recently, *A.alternata*, *A.tenuissima*, *A.burnsii*, and an unclassified species *Alternaria* sp. were identified as causal agents of leaf blight on maize in China (Xu et al. 2022). In rice, *A.padwickii* (Syn *Trichoconispadwickii*) has been frequently detected as a seed pathogen (Gutiérrez et al. 2010). Moreover, *A.arborescens* and *A.gaisen* were also reported as leaf spot pathogens of rice in Pakistan (Akhtar et al. 2014a, 2014b).

In this study, *Alternaria* spp. were isolated from symptomatic leaves of rice and maize in Guangxi Province and in Hainan Province in China, respectively. The aim of this study was to characterize these species taxonomically using morphological traits and multi-locus phylogenetic analysis.

## ﻿Materials and methods

### ﻿Isolation

In 2023, diseased maize (*Zeamays*) and rice (*Oryzasativa*) leaves exhibiting leaf spot and blight symptoms were collected in Guangxi and Hainan provinces, respectively. Leaf tissues were cut into small pieces with sterile blades and placed in petri dishes containing wet filter papers. After incubation at 25 °C for 1–2 days, fungal development on tissue samples were observed with a stereo microscope. Spores of *Alternaria* spp. developed from the edge of the leave tissues were singly picked using sterile glass needles and inoculated onto PDA (potato dextrose agar, Difco, Montreal, Canada). Pure cultures were deposited in the Fungi Herbarium of Yangtze University in Jingzhou, China. Dried cultures of the strains were also preserved in the herbarium for long-term storage.

### ﻿Morphology

Colony characteristics of strains of *Alternaria* spp. were observed and recorded following 7 days of incubation at 25 °C on 90-mm PDA plates under dark conditions. To determine their conidial morphology, the strains were grown on potato carrot agar (PCA) and V8 juice agar (V8A) at 25 °C for 7 days under a photoperiod of 8 hours of light per day. Conidia of the strains were observed and imaged with an ECLIPSE Ni-U optical microscope (Nikon, Tokyo, Japan). The dimensions of the conidia were measured (n = 50). Conidial morphology was determined based on sporulation pattern and conidial characteristics.

### ﻿PCR amplification

Fresh mycelia of the fungal strains grown on PDA were harvested and used for genomic DNA extraction following the procedures described by Watanabe et al. (2010). DNA solutions were then used to amplify fragments from several gene regions, including ITS, *GAPDH*, *RPB2*, *TEF1*, *Alt a 1*, *EndoPG*, and OPA10-2. Polymerase chain reaction (PCR) amplification of the above-mentioned regions was performed by a Bio-Rad T100^TM^ Thermal Cycler with primer pairs ITS5/ITS4 (White et al. 1990), gpd1/gpd2 (Berbee et al. 1999), EF1-728F/EF1-986R (Carbone and Kohn 1999), RPB2-5F/RPB2-7cR (Liu et al. 1999), Alt-for/Alt-rev (Hong et al. 2005), PG3/PG2b (Andrew et al. 2009), and OPA10-2L/OPA10-2R (Andrew et al. 2009), respectively. Reaction conditions for the PCR amplification were referred to previous studies (Woudenberg et al. 2015, Nwe et al. 2024). Successful amplification products were sent to TSINGKE (Beijing, China) for purification and Sanger sequencing in both directions. Sequences obtained from the company were manually examined with BioEdit v7.0.9 (Hall 1999) and then trimmed using MEGA X (Kumar et al. 2018). Consensus sequences were deposited in GenBank (https://www.ncbi.nlm.nih.gov/) with accession numbers shown in Table 1. New species are established based on the recommendations outlined by Jeewon and Hyde (2016).

**Table 1.** GenBank accession numbers of *Alternaria* spp. used for phylogenetic analysis.

Species	Strain	ITS	*Alt a 1*	*GAPDH*	*RPB2*	*TEF1*	OPA10-2	*EndoPG*
*A.alstroemeriae*	CBS 118808	KP124296	KP123845	KP124153	KP124764	KP125071	KP124601	KP123993
*A.alstroemeriae*	CBS 118809^T^	KP124297	–	KP124154	KP124765	KP125072	KP124602	KP123994
*A.alternantherae*	CBS 124392	KC584179	KP123846	KC584096	KC584374	KC584633	–	–
*A.alternata*	CBS 916.96^T^	AF347031	AY563301	AY278808	KC584375	KC584634	KP124632	JQ811978
*A.alternata*	CBS 112249	KP124338	KP123886	KP124192	KP124806	KP125114	KP124648	KP124039
*A.arborescens*	CBS 119544^T^	KP124408	KP123955	JQ646321	KP124878	KP125186	KP124722	KP124112
*A.arborescens*	CBS 102605^T^	AF347033	AY563303	AY278810	KC584377	KC584636	KP124712	AY295028
*A.arctoseptata*	MFLUCC 21-0139^T^	–	OK236755	OK236702	OK236655	OK236608	–	–
*A.baoshanensis*	MFLUCC 21-0124^T^	MZ622003	OK236760	OK236706	OK236659	OK236613	–	–
*A.betae-kenyensis*	CBS 118810^T^	KP124419	KP123966	KP124270	KP124888	KP125197	KP124733	KP124123
*A.breviconidiophora*	MFLUCC 21-0786^T^	MZ621997	OK236751	OK236698	OK236651	OK236604	–	–
*A.burnsii*	CBS 118816	KP124423	KP123970	KP124273	KP124892	KP125201	KP124737	KP124127
*A.burnsii*	CBS 118817	KP124424	KP123971	KP124274	KP124893	KP125202	KP124738	KP124128
*A.burnsii*	CBS 107.38^T^	KP124420	KP123967	JQ646305	KP124889	KP125198	KP124734	KP124124
*A.burnsii*	CBS 879.95	KP124422	KP123969	KP124272	KP124891	KP125200	KP124736	KP124126
*A.burnsii*	CBS 130264	KP124425	KP123972	KP124275	KP124894	KP125203	KP124739	KP124129
*A.burnsii*	CBS 110.50	KP124421	KP123968	KP124271	KP124890	KP125199	KP124735	KP124125
*A.burnsii*	CBS 108.27	KC584236	KP123850	KC584162	KC584468	KC584727	KP124605	KP123997
*A.eichhorniae*	CBS 489.92^T^	KC146356	KP123973	KP124276	KP124895	KP125204	KP124740	KP124130
*A.ellipsoidialis*	MFLUCC 21-0132	MZ621989	OK236743	OK236690	OK236643	OK236596	–	–
*A.eupatoriicola*	MFLUCC 21-0122	MZ621982	OK236736	OK236683	OK236636	OK236589	–	–
*A.falcate*	MFLUCC 21-0123	MZ621992	OK236746	OK236693	OK236649	OK236599	–	–
*A.gaisen*	CBS 118488^R^	KP124427	KP123975	KP124278	KP124897	KP125206	KP124743	KP124132
*A.gaisen*	CBS 632.93^R^	KC584197	KP123974	KC584116	KC584399	KC584658	KP124742	AY295033
*A.gossypina*	CBS 104.32^T^	KP124430	JQ646395	JQ646312	KP124900	KP125209	KP124746	KP124135
*A.gossypina*	CBS 102601	KP124433	KP123979	KP124282	KP124903	KP125212	KP124749	KP124138
*A.iridiaustralis*	CBS 118487	KP124436	KP123982	KP124285	KP124906	KP125215	KP124752	KP124141
*A.iridiaustralis*	CBS 118486^T^	KP124435	KP123981	KP124284	KP124905	KP125214	KP124751	KP124140
*A.jacinthicola*	CBS 878.95	KP124437	KP123983	KP124286	KP124907	KP125216	KP124753	KP124142
*A.jacinthicola*	CPC 25267	KP124439	KP123985	KP124288	KP124909	KP125218	KP124755	KP124144
*A.jacinthicola*	CBS 133751^T^	KP124438	KP123984	KP124287	KP124908	KP125217	KP124754	KP124143
*A.jingzhouensis*	YZU 221144^T^	OR883772	OR887694	OR887690	OR887688	OR887686	OR887684	OR887692
*A.koreana*	SPL2-1^T^	LC621613	LC631831	LC621647	LC621681	LC621715	LC631857	LC631844
*A.lathyri*	MFLUCC 21-0140^T^	MZ621974	OK236728	OK236675	OK236628	OK236581	–	–
*A.lijiangensis*	YZU 221458^T^	OQ679970	OQ686781	OQ686785	OQ686789	OQ686783	OQ686787	OQ686779
*A.longipes*	CBS 540.94^R^	AY278835	AY563304	AY278811	KC584409	KC584667	KP124758	KP124147
*A.longipes*	CBS 121332^R^	KP124443	KP123989	KP124292	KP124913	KP125222	KP124760	KP124149
*A.longxiensis*	YZU 221221^T^	OQ534546	OQ473629	OQ512732	OQ543009	OQ512726	OQ543003	OQ512720
*A.lycopersici*	YZU 221185^T^	OQ519795	OQ473633	OQ512736	OQ543013	OQ512730	OQ543007	OQ512724
*A.macilenta*	MFLUCC 21-0138^T^	MZ621972	OK236726	OK236673	OK236626	OK236579	–	–
*A.macroconidia*	MFLUCC 21-0134^T^	MZ622001	OK236757	OK236704	OK236657	OK236610	–	–
*A.minimispora*	MFLUCC 21-0127^T^	MZ621980	OK236734	OK236681	OK236634	OK236587	–	–
*A.momordicae*	YZU 161378^T^	OR883774	OR887695	OR887691	OR887689	OR887687	OR887685	OR887693
*A.muriformispora*	MFLUCC 21-0784^T^	MZ621976	OK236730	OK236677	OK236630	OK236583	–	–
*A.myanmarensis*	YZU 231736^T^	OR897031	OR979657	OR963612	PP508256	OR963615	PP034184	OR979663
*A.oblongoellipsoidea*	MFLUCC 22-0074^T^	MZ621967	OK236721	OK236668	OK236621	OK236574	–	–
*A.obpyriconidia*	MFLUCC 21-0121^T^	MZ621978	OK236732	OK236680	OK236633	OK236585	–	–
*A.orobanches*	MFLUCC 21-0137^T^	MZ622007	OK236763	OK236710	–	–	–	–
***A.oryzicola* sp. nov.**	**YZU 231199^T^**	**PQ812549**	**PV155522**	**PV155536**	**PV155548**	**PV155528**	**PV155542**	–
*A.ovoidea*	MFLUCC 21-0782^T^	MZ622005	–	OK236708	OK236661	OK236614	–	–
*A.phragmiticola*	MFLUCC 21-0125^T^	MZ621994	OK236749	OK236696	OK236649	OK236602	–	–
***A.poae* sp. nov.**	**YZU 231197^T^**	**PQ812551**	**PV155524**	**PV155538**	**PV155550**	**PV155530**	**PV155544**	**PV155532**
***A.poae* sp. nov.**	**YZU 231198**	**PQ812550**	**PV155523**	**PV155537**	**PV155549**	**PV155529**	**PV155543**	**PV155531**
*A.rostroconidia*	MFLUCC 21-0136^T^	MZ621969	OK236723	OK236670	OK236623	OK236576	–	–
*A.salicicola*	MFLUCC 22-0072^T^	MZ621999	OK236753	OK236700	OK236653	OK236606	–	–
*A.solanicola*	YZU 221189^T^	OQ534548	OQ473631	OQ512734	OQ543011	OQ512728	OQ543005	OQ512722
*A.tomato*	CBS 103.30	KP124445	KP123991	KP124294	KP124915	KP125224	KP124762	KP124151
*A.tomato*	CBS 114.35	KP124446	KP123992	KP124295	KP124916	KP125225	KP124763	KP124152
*A.torilis*	MFLUCC 14-0433^T^	MZ621988	OK236741	OK236688	OK236641	OK236594	–	–
*A.yamethinensis*	YZU 231739^T^	OR889008	OR979655	OR963610	PP179253	OR963614	PP034182	OR979661
***A.zeae* sp. nov.**	**YZU 231602^T^**	**PQ812548**	**PV155521**	**PV155535**	**PV155547**	**PV155527**	**PV155541**	–
***A.zeae* sp. nov.**	**YZU 231638**	**PQ812547**	**PV155520**	**PV155534**	**PV155546**	**PV155526**	**PV155540**	–
***A.zeae* sp. nov.**	**YZU 231640**	**PQ812546**	**PV155519**	**PV155533**	**PV155545**	**PV155525**	**PV155539**	–

### ﻿Phylogenetic analysis

Nucleotide sequences generated in this study were subjected to BLASTn (https://blast.ncbi.nlm.nih.gov/Blast.cgi, accessed on 31 October 2024) for similarity searches against the NCBI nucleotide database. Reference sequences of *Alternaria* spp. used for phylogenetic analysis were obtained based on recent publications (Woudenberg et al. 2015; Li et al. 2022, 2023; Romain et al. 2022; Aung et al. 2024; Nwe et al. 2024). Phylogenetic analysis was performed using the OFPT (One-click Fungal Phylogenetic Tool) program developed by Zeng et al. (2023). In brief, sequences of each genetic region were aligned by MAFFT v7.307 online version (Katoh and Standley 2013; Katoh et al. 2019) and then trimmed using TrimAI (Capella-Gutiérrez et al. 2009). Subsequently, nucleotide substitution models of each dataset were tested by ModelFinder (Kalyaanamoorthy et al. 2017) and the best-fit model for each dataset was selected based on the Bayesian information criterion (BIC). All the datasets were concatenated with partition information and then used for maximum likelihood (ML) and Bayesian phylogenetic analyses with software IQ-TREE (Nguyen et al. 2015) and Mrbayes 3.2.7 (Ronquist et al. 2012), respectively. In the ML analysis, 1,000 replicates were performed using bootstrap approximation. In the Bayesian inference (BI) analysis, a Markov Chain Monte Carlo (MCMC) algorithm was employed, involving four MCMC chains running for 50,000,000 generations with sampling every 100 generations. Posterior probabilities (PP) were estimated after discarding the first 25% of sampled tree as burn-in. The consensus BI tree was generated once the average standard deviation of split frequencies fell below 0.01.

## ﻿Results

### ﻿Phylogenetic analysis

**Figure 1.**
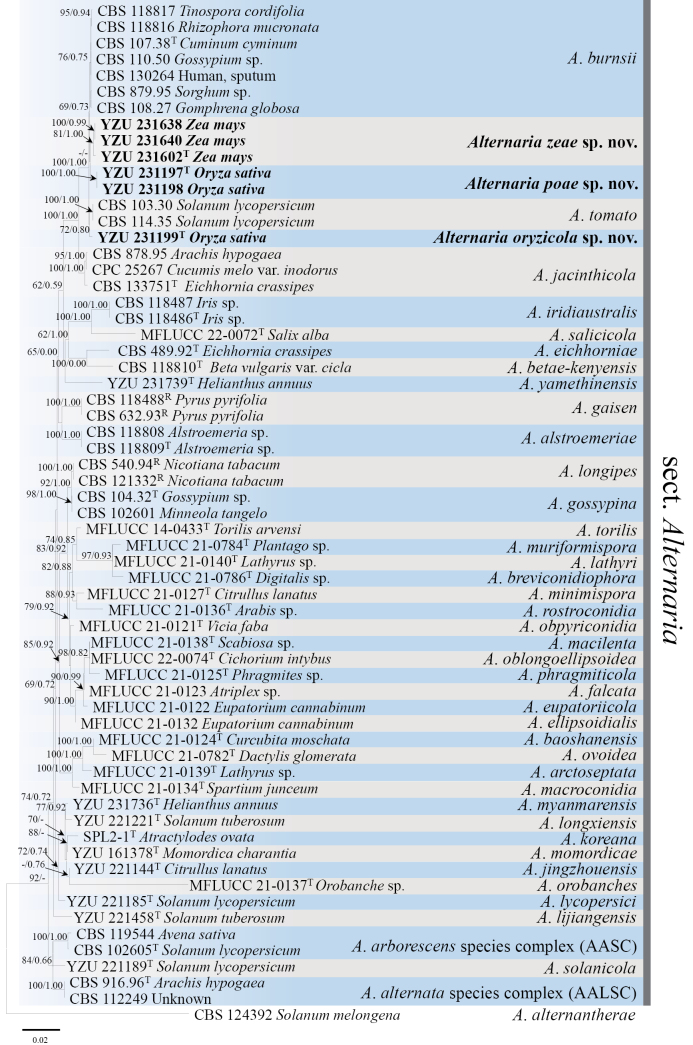
Phylogenetic tree constructed using the maximum likelihood method based on concatenated sequences of ITS, *GAPDH*, *RPB2*, *TEF1*, *Alt a 1*, *EndoPG*, and OPA10-2 from *Alternaria* spp. Bootstrap support values (BS) and Bayesian posterior probability (PP) are given at the nodes (BS/PP). The strains from this study are marked in bold. Ex-type strains are indicated with ‘T’, representative strains are indicated with ‘R’. *Alternariaalternantherae* CBS 124392 is used as the outgroup taxon.

A total of 63 strains (including 6 strains from this study) of Alternaria species in section Alternaria, were used for phylogenetic analysis. The concatenated sequence matrix consisted of seven loci, with a total length of 3608 bp, including 514 bp from ITS, 566 bp from *GAPDH*, 753 bp from *RPB2*, 234 bp from *TEF1*, 472 bp from *Alt a 1*, 448 bp from *EndoPG*, and 621 bp from OPA10-2. The best-fit evolutionary models for each gene were as follows: JC for ITS, TNe+G4 for *RPB2*, TIMe+I for *EndoPG*, TNe+R2 for OPA10-2, and K2P+G4 for *GAPDH*, *Alt a 1* and *TEF1*. In phylogenetic analyses, similar topologies were obtained from maximum likelihood and Bayesian methods. Additionally, the six strains examined in this study were placed within AlternariasectionAlternaria, clustering into three distinct clades. Specifically, strains YZU 231602, YZU 231638, and YZU 231640 (isolated from *Z.mays*) formed one clade supported with a bootstrap (BS) value of 81% and a Bayesian posterior probability (PP) of 1.00 (Fig. 1). This clade was positioned close to another clade composed of strains YZU 231197 and YZU 231198 (isolated from *O.sativa*). These two clades were relatively close to *A.burnsii* with BS/PP support values of 69%/0.73 (Fig. 1). Strain YZU 231199 isolated from *O.sativa* formed a single clade sister to strains of *A.tomato* (CBS 103.30 and CBS 114.35), supported with BS/PP values of 72%/0.80 (Fig. 1). The phylogenetic placements of these strains indicated that they represent three novel species in the genus AlternariasectionAlternaria.

### ﻿Taxonomy

#### ﻿ Alternaria oryzicola

Taxon classificationFungiPleosporalesPleosporaceae

H.F. Liu & J.X. Deng sp. nov.

B11107A8-0B7B-5431-A78F-D9D8DB8FE56D

857595

Fig. 2

##### Etymology.

Name refers to its host *Oryzasativa*.

##### Type.

China • Hainan Province, Lingshui County, diseased leaves of *Oryzasativa*, July 2023, J.L. Yin, holotype YZU-H-2023056A (permanently preserved in a metabolically inactive state), ex-type culture YZU 231199.

##### Description.

**Figure 2.**
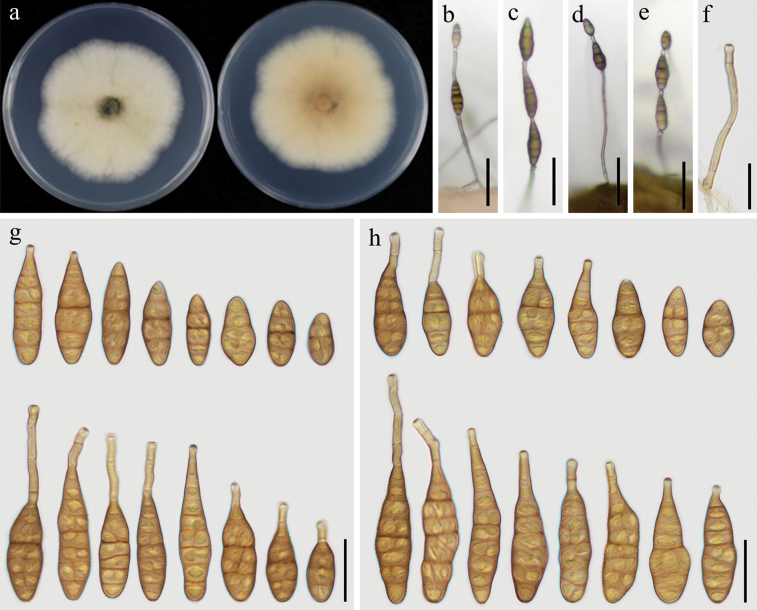
Morphology of *Alternariaoryzicola* sp. nov. (YZU 231199) **a** colony on PDA for 7 days at 25 °C **b, c** sporulation on PCA**d, e** sporulation on V8A**f** conidiophore and conidiogenous cell **g** conidia on PCA**h** conidia on V8A. Scale bars: 50 µm (**b, c, d, e**); 15 µm (**f**); 25 µm (**g, h**).

***Colonies*** on PDA sub-circular, velvety to fluffy, white to greyish-green, darker at the center, reverse side pale yellow to light brown, 61–63 mm in diameter (Fig. 2a). On PCA, ***conidiophores***, erect or curved, unbranched, sometimes slightly expanded at the apex, 32–105 × 3–4 μm in size, with 1–6 septa (Fig. 2f). ***Conidiogenous cells*** integrated, terminal, smooth, cylindrical, apically doliiform, 5–14 × 3–4 μm, with 1 conidiogenous locus. ***Conidia*** borne in chain, 1–3 units per chain, unbranched, mostly narrow-obclavate, obclavate, or long ellipsoid, 20–48 × 9–16 μm in dimension, 1–4 transverse septa, apical beak 4–39 × 2.5–4 μm (Fig. 2b, c, g). On V8A, ***conidiophores*** unbranched, 20–67 × 3–4 μm, with 1–5 septa. ***Conidia*** solitary or in chain with 2–3 units per chain, narrow-obclavate, obclavate, or long ellipsoid, 18–56 × 9–16 μm in size, with 2–6 transverse septa, apical beak 4–39 μm in length, 3–4 μm in width (Fig. 2d, e, h).

##### Notes.

Based on phylogenetic analysis using combined dataset of multiple regions, strain YZU 231199 was relatively close to strains of *Alternariatomato* (CBS 103.30 and CBS 114.35). Comparative analysis of nucleotide sequences revealed that strain YZU 231199 differed from representative strain of *A.tomato* (CBS 103.30) at four regions: 3 bp differences in *GAPDH* with 1 gap; 4 bp differences in *RPB2*, 1 bp difference in *TEF1*, and 1 bp difference in OPA10-2. Morphologically, the present fungus (YZU 231199) was also different with *A.tomato* in having smaller body size, less septa, and shorter beak (Table 2). Therefore, strain YZU 231199 was introduced as a novel species *A.oryzicola* sp. nov. in this study.

**Table 2.** Conidial morphology of *Alternaria* spp. from this study and previous publication.

Species	Conidia				Conidia per chain	Substrate	Reference
	Shape	Body size (μm)	Septa	Beak size (μm)			
*Alternariaburnsii*	ovoid or ellipsoid	30–50 × 9–13	5–8	–	Short chain	Host	Simmons (2007)
	narrow-ovoid or narrow-ellipsoid	30–40 × 8–14	3–7	–	–	PCA,V8A	Simmons (2007)
***A.oryzicola* sp. nov.**	**narrow-obclavate, obclavate, or long ellipsoid**	**20–48 × 9–16**	**1–4**	**4–39 × 2.5–4**	**1–3**	**PCA**	**This study**
		**18–56 × 9–16**	**2–6**	**4–39 × 3–4**	**1–3**	**V8A**	**This study**
***A.poae* sp. nov.**	**subellipsoid, obclavate, or narrow-ovoid**	**20–42 × 10–19**	**1–4**	**6–26 × 3–4**	**1–4**	**PCA**	**This study**
		**20–45 × 10–17**	**2–7**	**5–17 × 3–4**	**1–4**	**V8A**	**This study**
*A.tomato*	ellipsoid to long-ovoid	39–65 × 13–22	6–9	60–105 × 2	Solitary	Host	Simmons (2007)
***A.zeae* sp. nov.**	**ovate, ellipsoid or obclavate**	**26–46 × 10–18**	**3–6**	**9–93 × 2.5–4**	**1–4**	**PCA**	**This study**
		**26–45 × 10–17**	**3–6**	**4.5–65 × 2.5–4**	**1–4**	**V8A**	**This study**

#### ﻿ Alternaria poae

Taxon classificationFungiPleosporalesPleosporaceae

H.F. Liu & J.X. Deng sp. nov.

0FFDD089-97D7-5034-8E55-9AC177AD1C98

857596

Fig. 3

##### Etymology.

Name refers to its host family Poaceae.

##### Type.

China • Hainan Province, Lingshui County, diseased leaves of *Oryzasativa*, July 2023, J.L. Yin, holotype YZU-H-2023056B (permanently preserved in a metabolically inactive state), ex-type culture YZU 231197.

##### Description.

**Figure 3.**
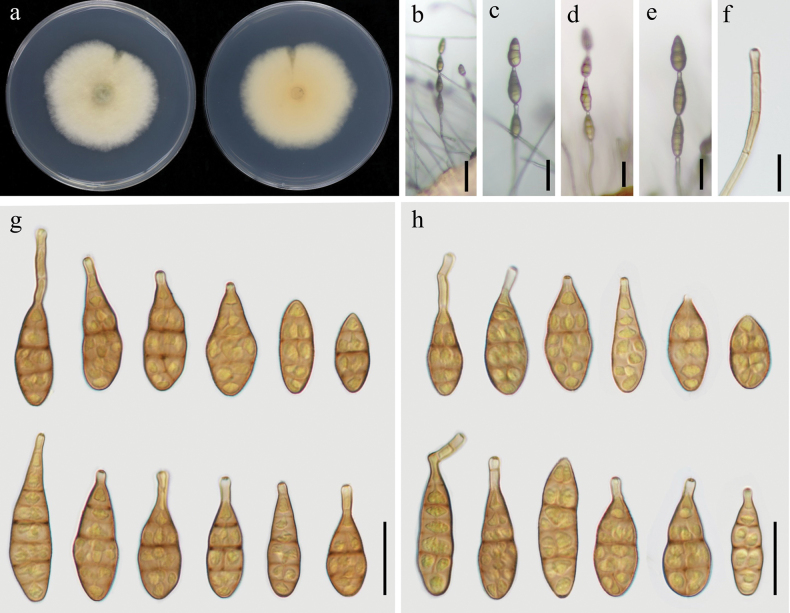
Morphology of *Alternariapoae* sp. nov. (YZU 231197) **a** colony on PDA for 7 days at 25 °C **b, c** sporulation on PCA**d, e** sporulation on V8A**f** conidiophore and conidiogenous cell **g** conidia on PCA**h** conidia on V8A. Scale bars: 50 µm (**b, c, d, e**); 15 µm (**f**); 25 µm (**g, h**).

On PDA, ***colonies*** sub-rounded, fluffy, cottony, white to pale green or yellow-green, reverse side pale yellow to light yellow, 55–56 mm in diameter (Fig. 3a). On PCA, ***conidiophores*** unbranched, curved or straight, 15–77 × 3–4 μm in size, with 1–5 septa (Fig. 3f). ***Conidiogenous cells*** 5–9 × 3–4 μm, integrated, terminal, cylindrical, thin-walled, smooth, apically doliiform, with 1 conidiogenous locus. ***Conidia*** borne single or in chain with at least 2–4 conidia per chain, unbranched, narrow-ovoid, subellipsoid, or obclavate, smooth, 20–42 × 10–19 μm, with 1–4 transverse septa. basal rounded, apical beak 6–26 × 3–4 μm (Fig. 3b, c, g). On V8A, ***conidiophores*** unbranched, smooth, 30–96 × 3–4 μm, with 2–7 septa. ***Conidia*** produced in chain with at least 2–4 conidia per chain, subellipsoid, obclavate, or narrow-ovoid, 20–45 × 10–17 μm, 1–4 transverse septa, beak 5–17 × 3–4 μm (Fig. 3d, e, h).

##### Additional isolated examined.

China • Hainan Province, Lingshui County, diseased leaves of *Oryzasativa*, July 2023, J.L. Yin, living culture YZU 231198.

##### Notes.

In phylogenetic analysis using concatenated sequences of ITS, *GAPDH*, *RPB2*, *TEF1*, *Alt a 1*, *EndoPG*, and OPA10-2, strains of *Alternariapoae* (YZU 231197 and YZU 231198) fell into a separate clade close to clades of *A.zeae* and *A.burnsii*. Based on nucleotide sequences, *A.poae* differs from *A.zeae* in five loci (3 bp in *GAPDH* with 1 gap, 5 bp in *RPB2*, 3 bp in *TEF1*, 3 bp in *Alt a 1*, and 7 bp in OPA10-2), and differs from *A.burnsii* in six loci (2 bp in *GAPDH*, 2 bp in *RPB2*, 3 bp in *TEF1*, 2 bp in *Alt a 1*, 2 bp in *EndoPG*, and 4 bp in OPA10-2). In morphology, *A.poae* can be distinguished from *A.zeae* by its shorter beak length, and from *A.burnsii* by its wider conidia bodies (Table 2).

#### ﻿ Alternaria zeae

Taxon classificationFungiPleosporalesPleosporaceae

H.F. Liu & J.X. Deng sp. nov.

18F932CA-2B12-51AD-AEA0-BE7BC7D9741B

857597

Fig. 4

##### Etymology.

Name refers to its host *Zeamays*.

##### Type.

China • Guangxi Province, Liuzhou City, diseased leaves of *Zeamays*, September 2023, F.Y Liu, holotype YZU-H-2023150A (permanently preserved in a metabolically inactive state), ex-type culture YZU 231602.

##### Description.

**Figure 4.**
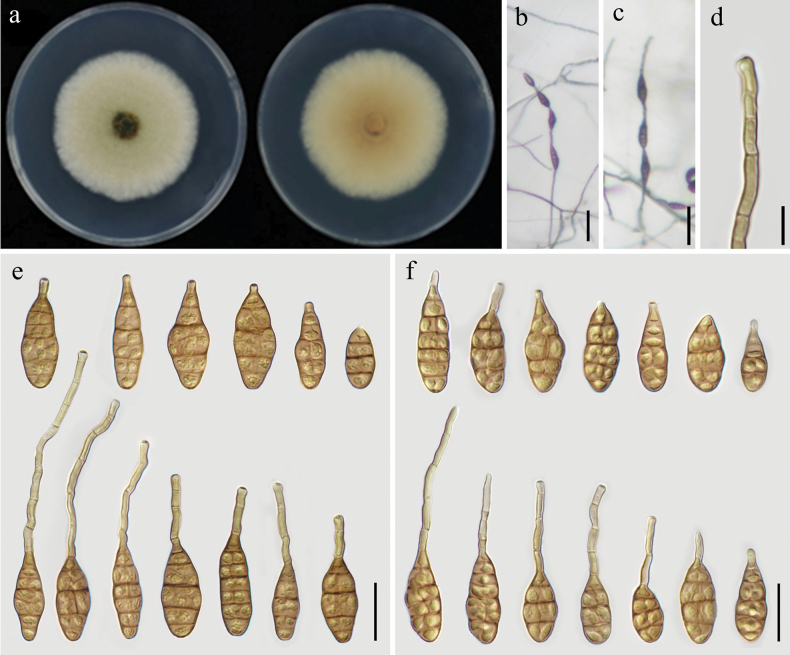
Morphology of *Alternariazeae* sp. nov. (YZU 231602) **a** colony on PDA for 7 days at 25 °C **b, c** sporulation on PCA**d** conidiophore and conidiogenous cell **e** conidia on PCA**f** conidia on V8A. Scale bars: 50 µm (**b, c**); 15 µm (**d**); 25 µm (**e, f**).

***Colonies*** on PDA round, fluffy, cottony, greenish-gray, white at the margin, reverse side pale yellow, 58–59 mm in diameter (Fig. 4a). The conidial morphology on PDA and PCA was similar, with only slight differences. On PCA, ***conidiophores*** straight or curved, unbranched, 25–123 × 2.5–4.5 μm, with 1–8 septa (Fig. 4d). ***Conidia*** borne singly or in chain with 2–4 conidia per chain, ovate, ellipsoid or obclavate, with 3–6 transverse septa, 26–46 × 10–18 μm in size, mostly with septate apical beak, 9–93 × 2.5–4 μm in size (Fig. 4b, e). On V8A, ***conidiophores*** straight or curved, unbranched, 38–118 × 2.5–4 μm, with 1–7 septa. ***Conidiogenous cells*** 5–14 × 3–5 μm, integrated, apical, cylindrical, light brown, smooth, apically doliiform, with 1 conidiogenous locus. ***Conidia*** solitary or produced in chain with 2–4 conidia, ovate, ellipsoid or obclavate, with 3–6 transverse septa, 26–45 × 10–17 μm, apical beak 4.5–65 × 2.5–4 μm, with 0–4 septa (Fig. 4c, f).

##### Additional isolates examined.

China • Guangxi Province, Liuzhou City, diseased leaves of *Zeamays*, September 2023, F.Y. Liu, living culture YZU 231638 and YZU 231640.

##### Notes.

Strains of *Alternariazeae* (YZU 231602, YZU 231638 and YZU 231640) formed a distinct clade in the multi-locus phylogenetic analysis. *Alternariapoae* and *A.burnsii* were genetically close to *A.zeae*. In nucleotide sequences, *A.zeae* differs from *A.poae* at five loci: 3 bp in *GAPDH* with 1 gap, 5 bp differences in *RPB2*, 3 bp in *TEF1*, 3 bp in *Alt a 1*, and 7 bp in OPA10-2. Nucleotide sequence differences were also observed between *A.zeae* and *A.burnsii* (3 bp in *GAPDH* with 1 gap, 2 bp in *RPB2*, 1 bp in *Alt a 1*, and 1 bp in OPA10-2). Morphologically, *A.zeae* has obviously longer beak than *A.poae* and *A.burnsii* (Table 2). In addition, conidia bodies of *A.zeae* are also wider than those of *A.burnsii* (Simmons 2007).

## ﻿Discussion

Based on integrated analyses of morphological characterization and multi-locus phylogenetic study, three novel species of *Alternaria* (*A.oryzicola* sp. nov., *A.poae* sp. nov., and *A.zeae* sp. nov.) from two different cereal crops (*O.sativa* and *Z.mays*) were described in this study. These findings contribute to the understanding of the diversity of *Alternaria* spp. on cereal crops in China.

In phylogenetic analysis using concatenated sequences of ITS, *GAPDH*, *RPB2*, *TEF1*, *Alt a 1*, *EndoPG*, and OPA10-2, all of the three novel species were assigned to distinct clades in AlternariasectionAlternaria. This section contains most of the small-spored species, which include important plant, human and postharvest pathogens (Woudenberg et al. 2015). Phylogenetically, species *A.zeae* sp. nov. and *A.poae* sp. nov. were relatively close to *A.burnsii* and *A.oryzicola* sp. nov. was relatively close to *A.tomato*. These species were located at the top of the phylogenetic tree of section Alternaria. In terms of morphology, the three species from this study were distinguished from their related species (*A.burnsii* and *A.tomato*) based on conidial characteristics, such as conidia size, septa, and beak size, as shown in Table 2. Therefore, both morphological and phylogenetic approaches provide evidence supporting the novelty of the species identified in this study.

In addition, the host is one of the important factors in the description of *Alternaria* species (Zhang 2003). According to fungus-host distribution in the USDA Fungal Databases (https://fungi.ars.usda.gov, accessed on 31 October 2024) and related publications, *A.burnsii* and *A.tomato* have been associated with different sources, but most are not from Poaceae plants. For example, *A.burnsii* was found on *Cuminumcyminum* (Simmons 2007; Woudenberg et al. 2015), *Tinosporacordifolia* (Woudenberg et al. 2015), *Rhizophoramucronata* (Woudenberg et al. 2015), *Gossypium* sp. (Woudenberg et al. 2015; El Gobashy et al. 2018), *Gomphrenaglobosa* (Woudenberg et al. 2015), *Sorghum* sp. (Kim et al. 2020), human sputum (Woudenberg et al. 2015), *Helianthusannuus* (Nwe et al. 2024), *Alliumcepa* (Htun et al. 2022), *Apiumgraveolens* (Zhuang 2005), *Buniumpersicum* (Mondal et al. 2002), *Cucurbitamaxima* (Paul et al. 2015), *Pandanus* sp. (Hyde et al. 2018), and *Zeamays* (Xu et al. 2022). *Alternariatomato* was reported on several plants, including *Solanumlycopersicum* (Simmons 2007), *Helianthusannuus* (Poudel et al. 2019), *Nopaleacochenillifera* (Infante et al. 2021), and *Phaseolusvulgaris* (Allen 1995). In the present study, the three novel species were isolated from two cereal crops (*Z.mays* and *O.sativa*), suggesting an increasing association of *Alternaria* species with Poaceae plants. According to previous studies, *Alternaria* spp. have been reported as predominant mycobiota in cereal grains (Kulik et al. 2015; Puvača et al. 2020; Orina et al. 2021). Most of these *Alternaria* species were predominantly classified in sections *Alternaria* and *Infectoriae* (Gannibal 2018), whereas some were sporadically found in section Pseudoalternaria (Gannibal 2018, Poursafar et al. 2018). Much attention has been devoted to detecting *Alternaria* spp. capable of producing mycotoxins (Orina et al. 2021). On cereal grains, several mycotoxins produced by *Alternaria* spp., such as AOH, AME, TEN, and TeA, were detected (Orina et al. 2021), posing potential risks to food safety. The ability of the three species in this study (*A.oryzicola* sp. nov., *A.poae* sp. nov., and *A.zeae* sp. nov.) to produce mycotoxins warrants further investigation. Since these three species were all isolated from diseased leaves of cereal crops, they could be potential pathogens. Furthermore, they are phylogenetically closely related to *A.burnsii* and *A.tomato*, which have recently been isolated and identified as pathogens of a cereal crop, wheat (Al-Nadabi et al. 2018).

Overall, this study characterized three novel species of *Alternaria* from two cereal crops, rice and maize, through morphological and molecular approaches. The potential interactions between these novel species and their host plants merit further investigation to uncover their ecological and agricultural impacts.

## Supplementary Material

XML Treatment for Alternaria oryzicola

XML Treatment for Alternaria poae

XML Treatment for Alternaria zeae

## ﻿Additional information

### Author ORCIDs

Hai-Feng Liu https://orcid.org/0000-0002-9733-9240

Feng-Yin Liu https://orcid.org/0000-0003-3114-603X

Jian-Xin Deng https://orcid.org/0000-0001-7304-5603

Hyunkyu Sang https://orcid.org/0000-0002-7459-5217

### Data availability

All of the data that support the findings of this study are available in the main text.
